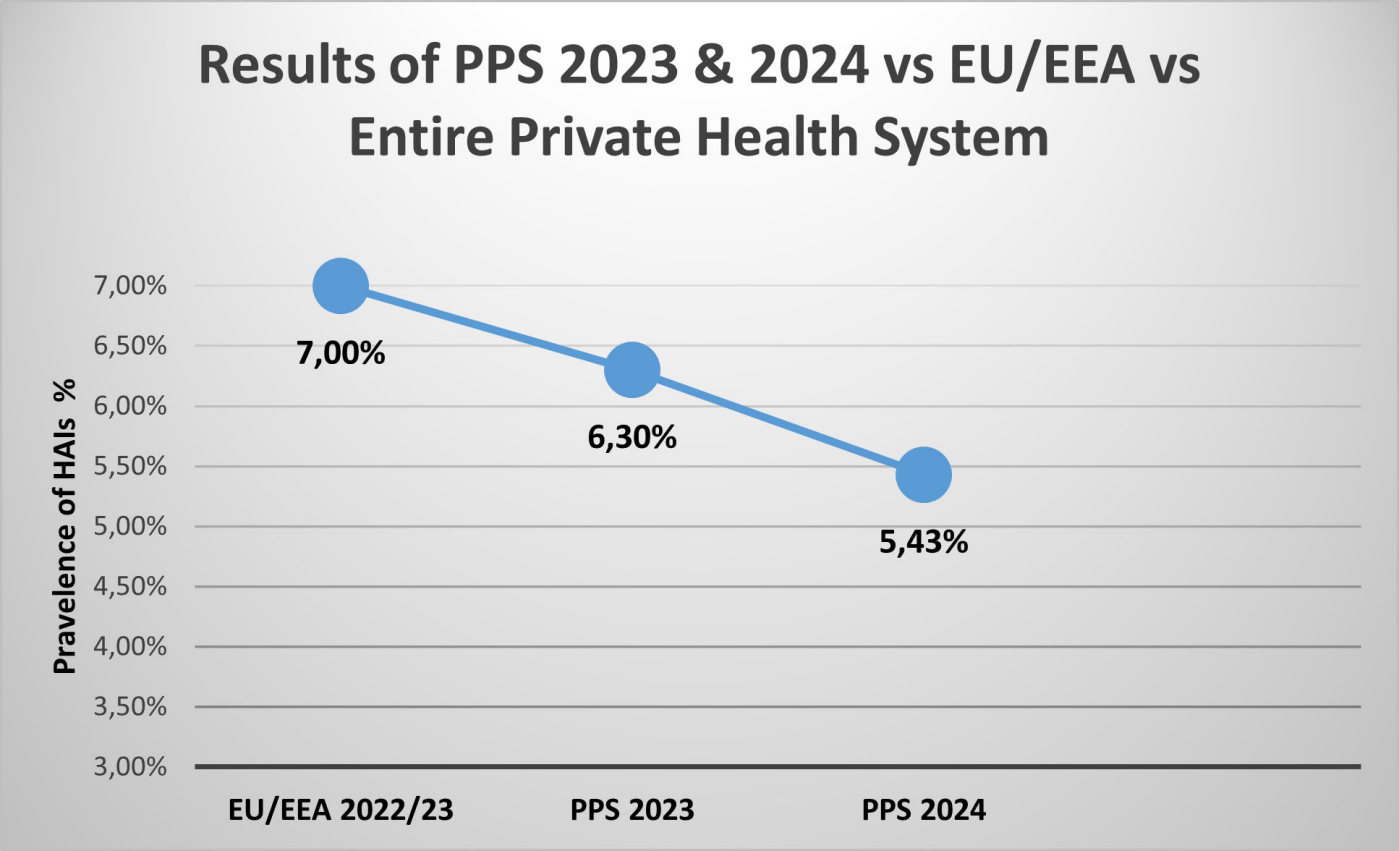# 248 Norovirus-Associated Acute Gastroenteritis Clusters in an Electronic Health Record System for Long-Term Care Facilities.

**DOI:** 10.1017/ash.2026.10618

**Published:** 2026-06-23

**Authors:** Tatiana Izakovic, Nikoleta Bartakovicova

**Affiliations:** 1 Hospital Bory, Bratislava, Penta Hospitals Slovakia

## Abstract

**Background:** There is an increased recognition of importance of quality of environmental cleaning services and its impact on hospital associated infections rates in healthcare facilities. In the era of growing prevalence of multidrug resistance organisms, a strong focus on high environmental services becomes even more important. From January 2024 to December 2024, our 400-bed acute care hospital with single patient room design located in capital of Slovakia has implemented a digital assessment tool of quality environmental cleaning services and measured its impact on hospital associated infections (HAI) rates. **Methods:** During a calendar year of 2024, two-hundred inpatient rooms were marked with fluorescent marker, fifty per quarter. These rooms were in adult intensive care unit and neonatal intensive care unit, general inpatient units, labor and delivery and mother and baby units. There were pre-defined 8 high touched surfaces marked in general inpatient rooms with 6 additional high touched surfaces in adjunct bathrooms and 10 high touched surfaces marked in intensive care unit’s patient rooms. The presence of fluorescent markings was evaluated using torch 24 hours after cleaning was performed and reported in digital tool downloaded on a mobile phone that provided almost real time data analysis of quality of cleaning based on the percentage of cleaned surfaces, by hospital, by units as well as by type of high touched surfaces. At the end of each quarter, results were discussed with units’ leadership and environment of care manager with quality improvement plan creation and implementation. Due to resource constraints, HAI surveillance relied on point prevalence surveys conducted in December 2023 and December 2024 rather than continuous incidence-based surveillance. **Results:** Our goal was to achieve 80% of effectively cleaned high touched surfaces, showing no residuals of fluorescent marking. The environmental cleaning quality improved substantially, with adequately cleaned surfaces increasing from 40% in first quarter to 82% in fourth quarter. HAI prevalence decreased from 6.30% (13/202 patients) in 2023 to 5.43% (11/206 patients) in 2024, representing a 0.87 percentage-point absolute reduction (13.8% relative decrease). This difference was not statistically significant (Fisher’s exact test, p ≈ 0.66). **Conclusions:** The limited sample size inherent to point prevalence methodology likely reduced statistical power and although the reduction in HAI prevalence did not reach statistical significance, we believe that the direction and magnitude of change, together with a marked improvement in environmental cleaning performance, proves a clinically meaningful reduction in HAI burden and increasement in patient safety.

**Figure:**
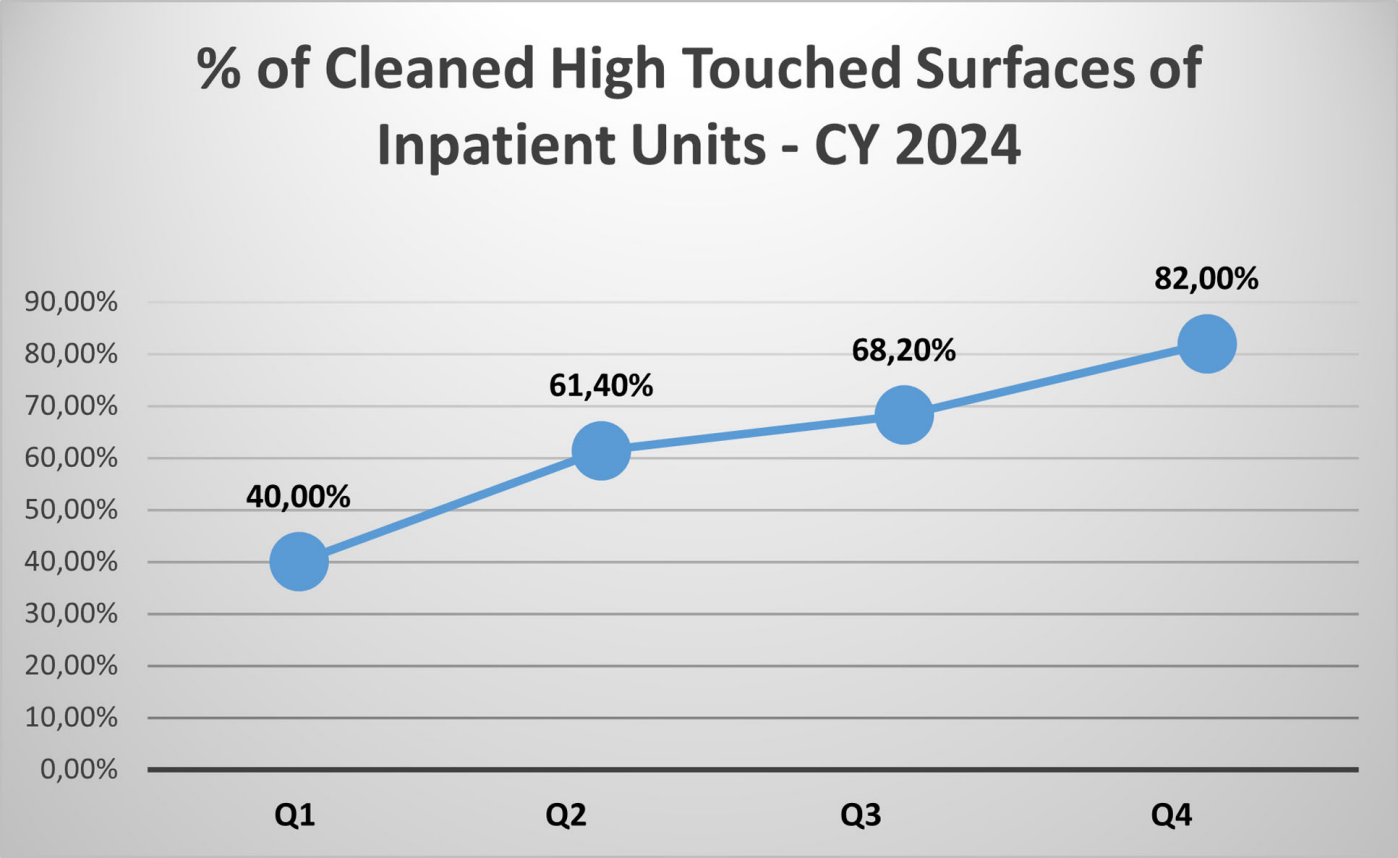

**Figure:**